# Mass Spectrometry Rearrangement Ions and Metabolic Pathway-Based Discovery of Indole Derivatives during the Aging Process in *Citrus reticulata* ‘Chachi’

**DOI:** 10.3390/foods13010008

**Published:** 2023-12-19

**Authors:** Tian Li, Ke Chen, Xiaoming Wang, Ying Wang, Yue Su, Yinlong Guo

**Affiliations:** 1 Institute of Interdisciplinary Integrative Medicine Research, Shanghai University of Traditional Chinese Medicine, 1200 Cailun Road, Shanghai 201203, China; yunnieedu@163.com; 2 State Key Laboratory of Organometallic Chemistry, Shanghai Institute of Organic Chemistry, Chinese Academy of Sciences, Shanghai 200032, China; chenke@sioc.ac.cn (K.C.); xiaoming@sioc.ac.cn (X.W.); ylguo@sioc.ac.cn (Y.G.); 3 Institute for Control of Chinese Traditional Medicine and Ethnic Medicine, National Institutes for Food and Drug Control, No. 31 Huatuo Road, Daxing District, Beijing 102629, China

**Keywords:** *Citrus reticulata* ‘Chachi’, methyl *N*-methyl anthranilate, mass spectrometry, 3-hydroxyindole, indole derivatives

## Abstract

The rapid analysis and characterization of compounds using mass spectrometry (MS) may overlook trace compounds. Although targeted analysis methods can significantly improve detection sensitivity, it is hard to discover novel scaffold compounds in the trace. This study developed a strategy for discovering trace compounds in the aging process of traditional Chinese medicine based on MS fragmentation and known metabolic pathways. Specifically, we found that the characteristic component of *C. reticulata* ‘Chachi’, methyl *N*-methyl anthranilate (MMA), fragmented in electrospray ionization coupled with collision-induced dissociation (CID) to produce the rearrangement ion 3-hydroxyindole, which was proven to exist in trace amounts in *C. reticulata* ‘Chachi’ based on comparison with the reference substance using liquid chromatography–tandem mass spectrometry (LC–MS/MS). Combining the known metabolic pathways of 3-hydroxyindole and the possible methylation reactions that may occur during aging, a total of 10 possible indole derivatives were untargeted predicted. These compounds were confirmed to originate from MMA using purchased or synthesized reference substances, all of which were detected in *C. reticulata* ‘Chachi’ through LC–MS/MS, achieving trace compound analysis from untargeted to targeted. These results may contribute to explaining the aging mechanism of *C. reticulata* ‘Chachi’, and the strategy of using the CID-induced special rearrangement ion-binding metabolic pathway has potential application value for discovering trace compounds.

## 1. Introduction

In recent years, liquid chromatography–mass spectrometry (LC–MS) has played an extremely important role in pharmaceutical [1,2] and food [3,4,5] analyses. Untargeted metabolomics [6,7,8] based on LC–MS is a widely unbiased method for detecting metabolites in biological samples [9]. Based on screening differential metabolites and analyzing their metabolic pathways, potential biomarkers can be identified; however, data processing is complex, sensitivity is limited, and some low-abundance compounds cannot be detected, limiting its application in discovering new biomarkers and understanding disease mechanisms. Targeted metabolomics [6,10,11] uses reference substances to detect specific metabolites that have already been noticed, which can effectively improve sensitivity and more accurately elucidate the relationships between metabolites and diseases. The analysis strategy [12] of integrating the univariate and multivariate correlation analysis approaches has been applied to discover tumor-tissue-derived metabolites in plasma samples, improving the accuracy of untargeted metabolomics screening for biomarkers. A novel integrated strategy [13] that employs chemical derivatization, LC–MS, and the molecular networking technique enabled the rapid recognition and identification of carboxylated gut microbiota–host co-metabolites with high sensitivity and selectivity, with 261 carboxylated metabolites detected in mouse feces. Comprehensive two-dimensional gas chromatography/time-of-flight mass spectrometry was applied for the characterization of the acidic fraction and complex hydrocarbons in cigarette smoke condensates. This method also can be used in many cases for mixtures with comparable complexity [14,15]. Targeted data-dependent acquisition based on the inclusion list of differential and pre-identified ions was developed to improve the data quality of untargeted metabolomics [16]. The transition from untargeted to targeted analysis strategies can significantly increase detection sensitivity, but it is difficult to discover novel scaffold compounds.

We have previously established a method [17] to simulate and predict the degradation products and metabolites of special drugs based on electrospray ionization–collision-induced dissociation–mass spectrometry (ESI–CID–MS). The acidic microdroplets [18] formed in ESI can accelerate degradation [19] and the metabolism of pharmaceuticals. Specifically, when an analyte is ionized and sprayed, it is charged and activated to become an excited species. The excited ions then enter the subsequent triple quadrupole and collide with Ar in the collision cell, undergoing non-specific free radical or rearrangement reactions, thereby enabling an accelerated process in the degradation and metabolism of drugs. Two low-abundance rearrangement ions, (aza) carbazole and (aza) biphenylene, were successfully identified using ESI–CID–MS of a series of commercially available kinase inhibitor drugs containing diphenylene-like structures. Thus, CID-induced rearrangement ions were crucial for discovering low-concentration compounds. Citri Reticulatae Pericarpium (CRP) is the dried pericarp derived from mature *Citrus reticulata* Blanco and its cultivars, which mainly include *C. reticulata* ‘Chachi’ from Guangdong, *C. reticulata* ‘Dahongpao’ from Sichuan, *C. reticulata* ‘Tangerina’ from Fujian, and *C. reticulata* ‘Unshiu’ from Zhejiang [20,21]. Among them, *C. reticulata* ‘Chachi’, known as ‘Xinhuichenpi’ and produced in Xinhui District, Guangdong, is considered a geoherb due to its superior quality, containing bioactive substances such as flavonoids [22,23], alkaloids [24], essential oils [25], and polysaccharides [26], and has extensive pharmacological effects. For example, polymethoxyflavones have anti-inflammatory effects [27,28], multiple anti-cancer effects [29,30,31], antiviral effects [23,32], and can also alleviate spleen deficiency and gastrointestinal diseases [33]. The alkaloid component, synephrine, has an anti-asthmatic effect [24]. In addition, as a traditional Chinese medicine with a long history, *C. reticulata* ‘Chachi’ is not only used in clinical practice but is also often used in food because of its high edible value. *C. reticulata* ‘Chachi’ needs to be stored for at least 3 to 10 years, and the longer the aging time, the darker the color and the better the quality. The process of the pericarp turning from yellow to brown or even black was attributed to the Maillard reaction in a number of studies [22]. With the aging of CRP, the total amounts of amino acids and reducing sugars in the substrate of the Maillard reaction decrease, further generating the intermediate 5-hydroxymethylfurfural. In the final stage of the Maillard reaction, various active intermediates undergo condensation and other reactions with amino acids to form brown nitrogen-containing polymers or copolymer melanin, eventually leading to browning [34]. One investigation showed that an increase in the nitrogenous compounds in *C. reticulata* ‘Chachi’ preserved for ten years was related to the Maillard browning product [35]; therefore, nitrogen-containing compounds in the trace amounts generated during the aging process deserve further exploration. MMA, a characteristic component of *C. reticulata* ‘Chachi’, is an important basis for identifying the authenticity of *C. reticulata* ‘Chachi’ [36]. MMA was first discovered in *Choisya ternata* Kunth (Rutaceae) [37] and has antibacterial and antifungal effects [38] as well as analgesic potential [39]. MMA is also believed to have effects on the central nervous system due to its citrus essential oil aroma [40], such as antianxiety- and antidepressant-like effects [41].

As mentioned earlier, the aging and browning of *C. reticulata* ‘Chachi’ are related to an increase in nitrogen-containing compounds, while current research on MMA mainly focuses on changes in its content during storage [42]. Therefore, during the continuous aging and drying processes of *C. reticulata* ‘Chachi’, the conversion products produced by this characteristic component have attracted our attention, which may be conducive to explaining the aging mechanism. The fragment ion *m*/*z* 134 was identified in the MS/MS spectrum of MMA (Figure S2), whose structure might be 3-hydroxyindole according to the chemical principles and properties of ESI–CID–MS-induced rearrangement. This study confirmed the production of 3-hydroxyindole in the degradation of MMA under LC–MS/MS analysis.

3-hydroxyindole is believed to play an important role, alongside indican, in the synthesis of indole alkaloids in *Isatis indigotica* (Chinese woad), which is a species with an ancient and well-documented history of use as an indigo dye and medicinal plant [43], and similar plants also include *Polygonum tinctorium* [44,45] and *Isatis tinctoria* (European woad), which a species in the same genus as *I. indigotica* [46,47,48]. Currently, two biosynthetic pathways of 3-hydroxyindole have been reported [49]. The first is via indole (an intermediate metabolite of the tryptophan biosynthetic pathway) under the catalysis of a monooxygenase [50,51], which is released by indican. However, 3-hydroxyindole is unstable and can be oxidized to isatin, which further dimerizes to indigo and indirubin [52]. Shintaro Inoue et al. [53] cloned a flavin-containing monooxygenase (PtFMO) gene from *P. tinctorium* and found that indigo was produced in the presence of tryptophan when recombinant PtFMO was expressed in E. coli. The co-expression of PtFMO with indoxyl β-D-glucoside synthase, which catalyzes the glucosylation of 3-hydroxyindole, brought about the formation of indican in E. coli. In other words, as an indigo plant, *P. tinctorium* hydrolyzes the released indican to generate 3-hydroxyindole [54], which spontaneously converts to indigo in the presence of oxygen or undergoes a glycosylation reaction to recover indican. Using the dry roots and leaves of *I. indigotica*, named ‘Ban-Lan-Gen’ (BLG, Isatidis Radix) and ‘Da-Qing-Ye’ (DQY, Isatidis Folium), respectively [55], Zou et al. [52] reported a LC–MS/MS quantitative method to determine indican, isatin, indirubin, and indigo in BLG and DQY. The different chemical components of BLG and DQY lead to differences in their pharmacological effects: indigo is often used for coloring and protecting the liver and for treating antibiosis, and indirubin has physiological properties that include anti-inflammatory [56] and anti-tumor effects [57] as well as a strengthening effect on the immune system [58]. Various indirubin derivatives with physiological activity have also been developed [59]. Based on this, focusing on the active metabolites and their differences is helpful for explaining the superiority of *C. reticulata* ‘Chachi’.

In this study, a rearrangement ion was generated from MMA, induced by ESI–CID, and was predicted to be 3-hydroxyindole. Referring to the reported pathway of 3-hydroxyindole, namely its oxidation and coupling and possible methylation reactions during the aging process of *C. reticulata* ‘Chachi’, compounds derived from MMA were untargeted predicted and derived. These trace compounds were then detected using targeted analysis to confirm their existence in *C. reticulata* ‘Chachi’, and their changes during the aging process were observed using LC–MS/MS. In summary, we aimed to develop a strategy for discovering trace compounds in the aging process of *C. reticulata* ‘Chachi’ based on MS fragmentation and known metabolic pathways, providing reference values for the analysis of trace compounds from untargeted speculation to targeted verification.

## 2. Materials and Methods

### 2.1. Reagents and Materials

MMA was purchased from Zesheng Technology Co., Ltd. (Anhui, China) and 3-hydroxyindole was obtained from J&K Scientific (Shanghai, China). Isatin, indigo, and indirubin were purchased from Beijing Innochem Technology Co., Ltd. (Beijing, China). MDA was purchased from TCI (Shanghai, China). *N*-methylisatin was purchased from Bide Pharmatech Ltd. (Shanghai, China). Dimethyl sulfoxide (DMSO) was purchased from Solarbio (Beijing, China). Methanol, acetonitrile, and ethanol (LC–MS grade) were obtained from Merck (Darmstadt, Germany). Water was purified with a Milli-Q water-purification system (Millipore Corp., Bedford, MA, USA).

### 2.2. Synthesis of Compounds ***7***, ***9***, ***11***, and ***12***

**Scheme 1:**
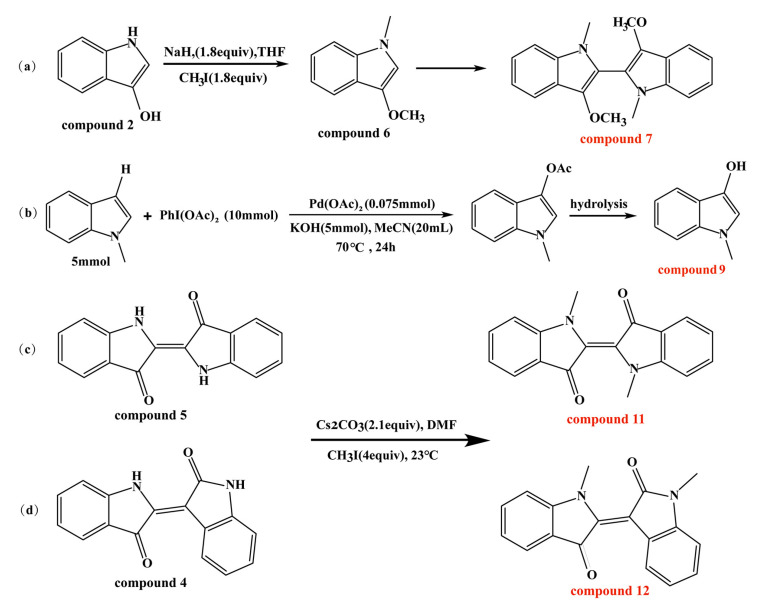
(**a**–**d**) Approaches for synthesis of compounds **7, 9**, **11**, and **12**.

*N*, *N*′, *O*, *O*′-Tetramethyl-leuko-indigo [60,61], *N*-methyl-3-hydroxyindole [62], *N*, *N*′-demethylindigo, and *N*, *N*′-demethylindirubin [63,64] were synthesized according to the reported schemes (Scheme 1). The specific synthesis steps and the results of NMR and liquid chromatography–high-resolution tandem mass spectrometry (LC–HR–MS) are all described in the Supplementary Materials (Figure S1).

### 2.3. Collection of C. reticulata ‘Chachi’ and C. reticulata ‘Dahongpao’

*C. reticulata* ‘Chachi’ fruits aged for different years were obtained from Xinhui District, Jiangmen City, and Guangdong Province, and were authenticated by the National Institutes for Food and Drug Control. *C. reticulata* ‘Dahongpao’ samples were obtained from Sichuan Province. Detailed sample information is listed in Table S1.

### 2.4. Samples Extraction

Each sample of *C. reticulata* ‘Chachi’ and *C. reticulata* ‘Dahongpao’ was ground to a powder with the aid of a machine manufactured by Shanghai one bio Technology Co., LTD (Shanghai, China). An aliquot (1.0 g) of each sample was added to 20 mL of methanol with ultrasonication (SB-25-12DTD, Ningbo Scientz Biotechnology Co. Ltd., Ningbo, China) for 40 min at room temperature followed by centrifugation at 8000 rpm for 5 min. The supernatants were stored at −80 °C until analysis and filtered through a 0.22 μm membrane filter before direct injection into the LC–MS/MS system.

### 2.5. Preparation of Standard Solutions

Standard stock solutions of MMA (1 mg∙mL^−1^) and MDA (1 mg∙mL^−1^) were prepared in ethanol. Standard stock solutions of isatin (1 mg∙mL^−1^) and *N*-methylisatin (1 mg∙mL^−1^) were prepared in acetonitrile. Standard stock solutions of 3-hydroxyindole, indirubin, indigo, and their *N*,*N*-methylated derivatives were dissolved in DMSO to make a 1 mM stock solution, which was stored at −20 °C in the dark. The working standard solutions were prepared by diluting their stock solution with acetonitrile to a concentration of 100 μg∙mL^−1^ for the LC–HR–MS analysis and 1 μg∙mL^−1^ for the ESI–CID–MS analysis.

### 2.6. LC Conditions

Chromatographic separation was performed at 25 °C on a Thermo Scientific™ Hypersil GOLD™ Phenyl reversed-phase HPLC column (1.9 μm, 2.1 × 100 mm). A gradient elution of 0.1% formic acid in water (A) and acetonitrile (B) was used: 0–0.75 min, 5% B; 0.75–1 min, 5–25% B; 1–3 min, 25–50% B; 3–9 min, 50–95% B; 9–11 min, 95% B; 11–13 min, 95–5% B; and 13–15 min, 5% B. The injection volume was 3 μL and the flow rate was 0.2 mL/min.

### 2.7. MS Conditions

LC–HR–MS analysis was performed on the 1260 HPLC system and a quadrupole time-of-flight mass spectrometer equipped with an ESI interface (6538, Agilent). The mass spectrometer was operated in positive mode. The ESI parameters were optimized as follows: capillary voltage = 3000 V, drying gas (N_2_) = 5.0 L/min, gas temperature = 300 °C, nebulizer gas pressure = 40 psi, sheath gas (N_2_) = 10 L/min, and fragmentor = 150 V.

LC–ESI–CID–MS analysis was performed on a Waters Xevo TQ-S (Waters Corp., Milford, MA, USA), and the data were analyzed using MassLynx. The operating parameters were optimized as follows: capillary voltage = 3 kV, cone voltage = 65 kV, desolvation temperature = 350 °C, desolvation gas flow = 550 L/Hr, and collision gas flow = 0.15 mL/min. The collision energy of MMA, isatin, indigo, indirubin, and MDA was at 25 eV. The collision energy of 3-hydroxyindole, *N*, *N*′, *O*, *O*′-tetramethyl-leuko-indigo, *N*-methyl-3-hydroxyindole, *N*-methylisatin, *N*, *N*′-demethylindigo, and *N*, *N*′-demethylindirubin was at 20 eV.

## 3. Results

### 3.1. Prediction of Indole Derivatives Based on Rearrangement Ions and Metabolic Pathways

Based on previous speculation, the *m*/*z* 134 produced by MMA (compound **1**) may be 3-hydroxyindole (compound **2**). Meanwhile, isatin (compound **3**) has been reported to form after the oxidation of 3-hydroxyindole, further generating indigo (compound **4**) and indirubin (compound **5**) [46,52,65]. Therefore, the pathway from compound **1** to compound **5** was reasonably proposed.

In plants, modifying enzymes of different families can act on alkaloids to produce a variety of alkaloid derivatives having altered physical, chemical, and biological properties. The chemical modifications catalyzed by these enzymes mainly include methylation, glycosylation, oxidation, reduction, hydroxylation, and acylation [66]. Methylation reactions play a central role in the functionalization of specialized metabolites, and the methyl transferase (MT) gene family enzymes are the key substances responsible for catalyzing this type of reaction [67], such as the *O*-methyltransferase (OMT) and *N*-methyltransferase (NMT). OMT is the largest class of enzymes catalyzing methyl transfer at the oxygen atom position of alkaloid substrates; unlike OMT, NMT acts on the nitrogen atom of substrates [68,69]. For example, 6-*O*-methylated was the derivative of coclaurine through 1-benzylisoquinoline 6-OMT, then *N*-methylcellulose can be formed under the catalysis of *N*-methyltransferase in benzylisoquinoline alkaloid biosynthesis in sacred lotus (*Nelumbo nucifera*) [69]. Therefore, we speculated that the characteristic component MMA in *C. reticulata* ‘Chachi’ may undergo methylation during the ripening and drying process of *Citrus reticulata* Blanco, resulting in MDA (compound **8**), which is based on MMA with an additional methyl atom on the N atom. Similarly, MDA may also produce a structure of *m*/*z* 148, namely *N*-methyl-3-hydroxyindole (compound **9**), which was inferred to also undergo oxidation and coupling reactions, generating *N*-methylisatin (compound **10**), *N*, *N*′-demethylindigo (compound **11**), and *N*, *N*′-demethylindirubin (compound **12**). A production pathway from compound **8** to compound **12** may exist in *C. reticulata* ‘Chachi’ or other plants, corresponding to the pathway of compounds **1**–**5**.

**Figure 1:**
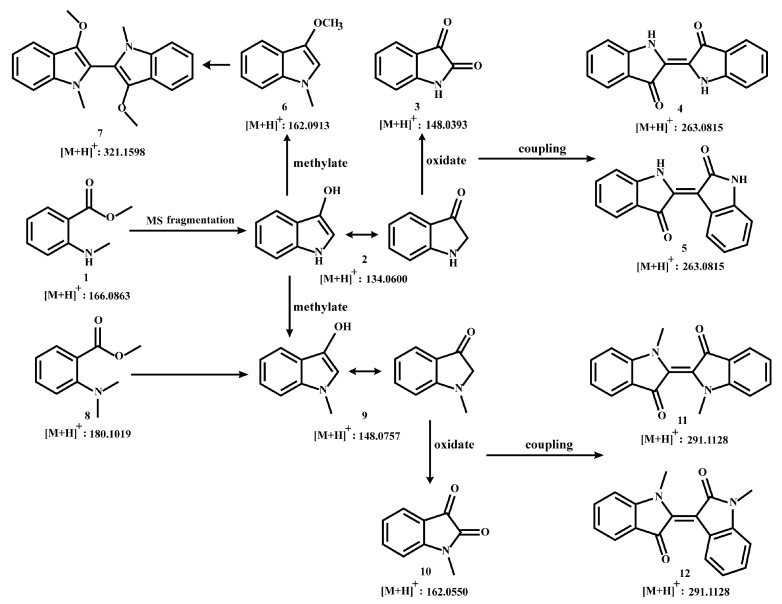
Putative diagram of indole derivatives.

In addition, compound **9** is a *N*-methylated derivative of compound **2**, and *O*-methylation reactions may also occur during the drying process of *C. reticulata* ‘Chachi’. Thus, it was predicted that compound **6** could be derived from compound **2** and coupled as a two-site complex. The speculated coupling product is *N*, *N*′, *O*, *O*′-tetramethyl-leuko-indigo (compound **7**). Based on the above speculation, we have summarized a production pathway of indole derivatives as shown in Figure 1.

### 3.2. Feasibility Verification of Predicted Compounds by the Reference Substance

**Figure 2:**
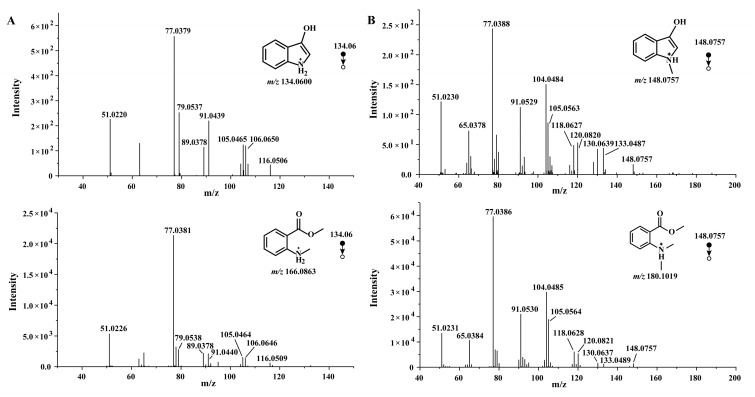
(**A**) MS/MS spectra of MMA and 3-hydroxyindole at *m*/*z* 134.06 using LC–HR–MS. (**B**) MS/MS spectra of MDA and N-methyl-3-hydroxyindole at *m*/*z* 148.0757 using LC–HR–MS.

Firstly, we validated that the fragment ion 134 generated by MMA was 3-hydroxyindole. The LC–HR–MS analysis accurately reflected the consistency between the 134.06 secondary fragment ions of MMA and 3-hydroxyindole (Figure 2A). According to the change from compound **1** to compound **2** in *C. reticulata* ‘Chachi’ during the aging process, *N*-methyl-3-hydroxyindole should be found by the same fragmentation mode. Similarly, we compared the 148.07 secondary fragment ions of MDA and the compound **9** reference substances using LC–HR–MS (Figure 2B).

The 3-hydroxyindole powder was directly exposed under ultraviolet light for 48 h and then analyzed using LC–HR–MS. The full scan spectra of the LC–HR–MS analysis revealed that *m*/*z* 148.0393 and *m*/*z* 263.0817 were produced after exposure to oxygen and UV irradiation, consistent with the reported pathways, matching with the [M+H] + of isatin, indigo, and indirubin, respectively, as shown in Table 1. The extracted ion chromatograms (EICs) and secondary fragment ions of the oxidized 3-hydroxyindole for protonated molecular weights at *m*/*z* 148.0 and *m*/*z* 263.1 further confirmed the production of known pathway products. The experimental phenomena during the synthesis process showed that *N*-methyl-3-hydroxyindole (under argon protection) was light green but, once exposed to oxygen, it oxidized and changed to dark green, indicating that other transformations occurred. Therefore, *N*-methyl-3-hydroxyindole at *m*/*z* 148.0757, *N*-methylisatin at *m*/*z* 162.0551, and *N*, *N*′-demethylindigo or *N*, *N*′-demethylindirubin at *m*/*z* 291.1128 were detected in synthetic solution using LC–HR–MS. The retention time of the EIC and secondary fragment ions at *m*/*z* 162.1 and *m*/*z* 291.1 were also consistent with compounds **10**, **11**, and **12**. As we speculated, *N*-methyl-3-hydroxyindole spontaneously oxidized and dimerized after coming in contact with air, similar to the oxidative dimerization pathway of 3-hydroxyindole.

In the LC–HR–MS spectrum of 3-hydroxyindole methylation, although 3-methoxy-1-methylindole at *m*/*z* 162.0913 was not found, it was observed at *m*/*z* 291.1128 and *m*/*z* 321.1592, which was consistent with our prediction. In other words, after methylation at both the N and O sites of 3-hydroxyindole, a two-site coupling occurred, forming *N*, *N*′, *O*, *O*′ -tetramethyl-leuko-indigo.

### 3.3. Analysis and Validation of Compounds in C. reticulata ‘Chachi’

**Figure 3:**
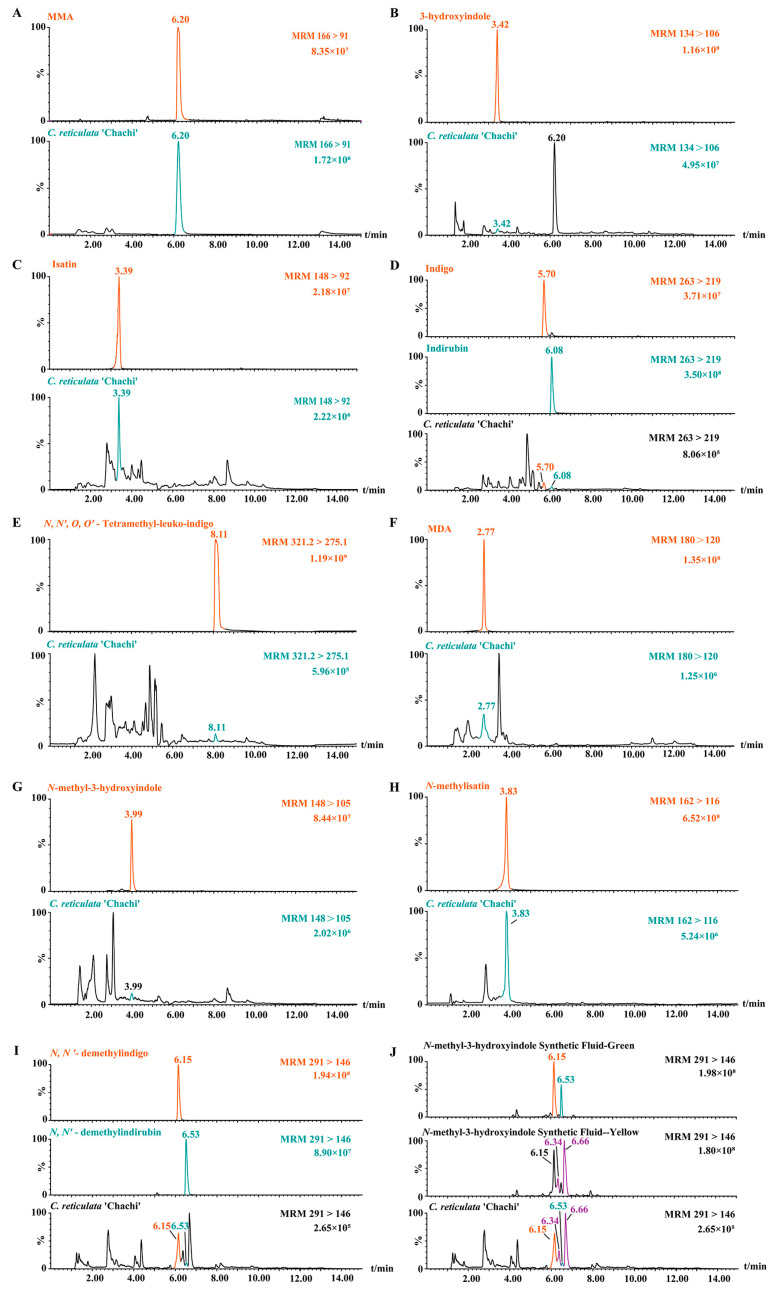
Comparison of MRM chromatograms between compounds **1**–**12** and *C. reticulata* ‘Chachi’. (**A**) MMA; (**B**) 3-hydroxyindole; (**C**) isatin; (**D**) indigo and indirubin; (**E**) *N*, *N*′, *O*, *O*′-Tetramethyl-leuko-indigo; (**F**) MDA; (**G**) *N*-methyl-3-hydroxyindole; (**H**) *N*-methylisatin; (**I**) *N*, *N*′-demethylindigo and *N*, *N*′-demethylindirubin; and (**J**) synthesis fluid of *N*-methyl-3-hydroxyindole.

Since MMA is a characteristic component in *C. reticulata* ‘Chachi’, the predicted compounds originating from MMA should be found in *C. reticulata* ‘Chachi’. This section examines the existence of the 11 compounds mentioned above in *C. reticulata* ‘Chachi’ using the multiple reaction monitoring (MRM) mode, except for 3-methoxy-1-methylindole because of the absence of a reference substance. MMA is known to be a characteristic component in *C. reticulata* ‘Chachi’, with an ion peak at 6.20 min (Figure 3A). The *m*/*z* 134→106 ion pairs of 3-hydroxyindole were selected for MRM analysis, showing its peak at 3.42 min. In the chromatogram of *C. reticulata* ‘Chachi’, although the main peak was at the peak position of MMA (Figure S3), an ion peak at 3.42 min was also observed, indicating the presence of 3-hydroxyindole (Figure 3B). This confirmed, for the first time, that MMA could be converted into 3-hydroxyindole in *C. reticulata* ‘Chachi’.

As shown in Figure 3C–I, the MRM spectra indicated that *C. reticulata* ‘Chachi’ and the compounds **3**–**12** have ion peaks at the same retention time. For example, using the rapid LC–MS/MS analysis of the *N*-methyl-3-hydroxyindole synthesized solution, the chromatogram of *m*/*z* 148→105 ion pairs showed that the retention time of *N*-methyl-3-hydroxyindole was 3.99 min, which is the same as that detected in *C. reticulata* ‘Chachi’ (Figure 3G). The retention time of, and secondary fragment ions between, *C. reticulata* ‘Chachi’ and compounds **1**–**12** were also broadly consistent, as shown in Table S2. The experimental results confirmed that our speculated production pathway of indole derivatives can be found not only in the reference substances but also in *C. reticulata* ‘Chachi’, which demonstrates the unique role of MMA in *C. reticulata* ‘Chachi’ and, for the first time, confirms that 3-hydroxyindole can convert into indole derivatives in *C. reticulata* ‘Chachi’.

The synthesis solution of *N*-methyl-3-hydroxyindole turned dark green after oxidation, and the color changed to yellow after 48 h. Correspondingly, the MRM chromatogram of the *m*/*z* 291→146 ion pairs showed that it yielded four ion peaks except for the peaks of *N*, *N*′-demethylindigo and *N*, *N*′-demethylindirubin (Figure 3J). Interestingly, *C. reticulata* ‘Chachi’ showed not only compounds **11** and **12** but also two other ion peaks at 6.34 min and 6.66 min, indicating that there may also be isomers of compounds **11** or **12** in *C. reticulata* ‘Chachi’, which requires further confirmation.

*C. reticulata* ‘Chachi’ fruits with different years of storage were collected, and the above compounds were analyzed successively, as summarized in Table 2. MMA, MDA, isatin, indigo, and indirubin were all detected, regardless of the storage time of *C. reticulata* ‘Chachi’. However, methylated products and their subsequent coupling products were not detected in some samples. With a storage period of 5–9 years, all of the compounds in the pathway were detected, indicating that the indole derivatives of *C. reticulata* ‘Chachi’ might be relatively stable during the aging time. *N*-methylsatin, *N*, *N*′-demethylindigo, and *N*, *N*′-demethylindirubin were not detected in the *C. reticulata* ‘Chachi’ samples stored for more than 10 years, which is meaningful for exploring the relationship between the efficacy and aging time of *C. reticulata* ‘Chachi’. Interestingly, MMA and 3-hydroxyindole were not found in *C. reticulata* ‘Dahongpao’, while small amounts of indirubin, MDA, and *N*-methyl-3-hydroxyindole were found (Figure S4). Therefore, it is considered that the types of indole derivatives are related to the colors of these two different types of cultivars; the color of *C. reticulata* ‘Chachi’ changed from orange to brown and, finally, to dark brown and even black during the drying and aging process, whereas *C. reticulata* ‘Dahongpao’ appears reddish in color.

## 4. Conclusions

A strategy for discovering trace compounds in the aging process of *C. reticulata* ‘Chachi’ was developed through the occurrence of non-specific free radical reactions or the rearrangement reaction in MS and known metabolic pathways. A total of 11 degradation products and metabolites were predicted and derived. In detail, the component 3-hydroxyindole was found in the fragment ions of the unique component MMA in *C. reticulata* ‘Chachi’, and the same fragmentation mode was found in MDA, the methylated product of MMA, which produced *N*-methyl-3-hydroxyindole. In addition, the oxidative dimerized pathway of 3-hydroxyindole can also occur in *N*-methyl-3-hydroxyindole, producing isatin, indigo, indirubin, and their *N*-methyl or *N*, *N*-dimethyl products, respectively. The LC–MS/MS detection of 11 compounds in different cultivated samples showed that MMA, 3-hydroxyindole, indigo, *N*, *N*′-demethylindigo, and *N*, *N*′-dimethylindirubin as well as *N*, *N*′, *O*, *O*′- tetramethylindigo were only found in *C. reticulata* ‘Chachi’. Isatin, indirubin, MDA, *N*-methyl-3-hydroxyindole, and *N*-methylisatin were also found in the reddish *C. reticulata* ‘Dahongpao’ cultivar. Further study is needed of the reasons for the differences in these indole compounds between the two cultivars. Moreover, these 11 indole derivatives could be detected in *C. reticulata* ‘Chachi’ during a storage period of 5–9 years, while some substances could not be detected after 10 years. This change was conducive to explaining the age and browning color of *C. reticulata* ‘Chachi’. In addition, there might also be isomers in the *C. reticulata* ‘Chachi’, except for *N*, *N*′-demethylindigo and *N, N’*-demethylindirubin. The presence of these indole derivatives was reported for the first time in *C. reticulata* ‘Chachi’, which is of great significance for explaining the aging process and superiority of *C. reticulata* ‘Chachi and might be applied to distinguish species. The described strategy expands the method for discovering low-abundance compounds in drugs, and more comprehensively explores trace compounds in drugs, to explain their relationships with pharmacological activity.

## Figures and Tables

**Table 1:** Feasibility verification of predicted compounds using the reference substances.

Substrates	Reaction	Products	Structure	Retention Time/min	[M+H]^+^		Secondary Fragment Ions (*m*/*z*)
					Theoretical Mass (*m*/*z*)	Experimental Mass (*m*/*z*)	
3-hydroxyindole	oxidation	Isatin	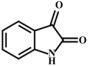	3.39	148.0393	148.0393	65.0, 77.0, 92.0, 102.0, 120.0, 130.0
		Indigo	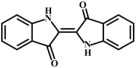	5.70	263.0815	263.0817	132.0, 206.0, 180.0, 190.0, 235.0, 219.0
		Indirubin	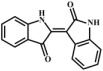	6.08	263.0815	263.0817	190.0, 219.0, 165.0, 132.0, 235.0, 206.0
	methylation	*N*, *N*′, *O*, *O*′-tetramethyl-leuko-indigo	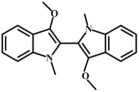	8.11	321.1598	321.1592	306.1, 291.1, 275.1, 247.1, 175.1, 160.1
*N*-methyl-3-hydroxyindole	oxidation	*N*-methylisatin	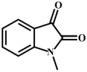	3.83	162.0550	162.0551	116.0, 106.0, 79.0, 65.0, 77.0, 134.0, 144.0
		*N*, *N*′-demethylindigo	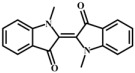	6.15	291.1128	291.1128	132.0, 276.0, 146.1, 247.1, 120.1, 172.1
		*N*, *N*′-demethylindirubin	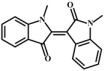	6.53	291.1128	291.1128	247.1, 276.1, 146.1, 132.0, 263.1, 158.1

Note: the retention time, accurate mass, and secondary fragment ions were consistent with the reference substances.

**Table 2:** Differences between 12 compounds of *C. reticulata* ‘Chachi’ with different storage times and *C. reticulata* ‘Dahongpao’.

Samples Number	Aging Time	MMA	3-Hydroxyindole	Isatin	Indigo	Indirubin	*N*, *N*′, *O*, *O*′-tetramethyl-leuko-indigo	MDA	*N*-methyl-3-hydroxyindole	*N*-methylisatin	*N*, *N*′-demethylindigo	*N*, *N*′-demethylindirubin
1	1	+++	−	++	+	+	+	++	++	++	+	+
2	2	+++	++	++	+	+	−	++	++	−	+	+
3	2	+++	−	++	++	+	+	++	−	++	+	+
4	3	+++	++	++	+	+	++	++	++	−	+	+
5	3	+++	−	++	+	+	−	++	+++	++	−	+
6	3	+++	++	++	+	+	+	++	++	++	+	−
7	5	+++	++	++	+	+	+	++	++	++	+	+
8	5	+++	++	++	+	+	+	++	++	++	+	+
9	6	+++	++	++	+	+	+	++	++	++	+	+
10	9	+++	++	++	+	+	+	++	++	++	+	+
11	10	+++	++	++	+	+	−	++	++	++	++	+
12	10	+++	++	++	+	+	+	++	++	−	−	+
13	>10	+++	++	++	+	+	+	++	++	−	+	+
14	>10	+++	++	++	+	+	+	++	++	−	−	+
15	>10	+++	++	++	+	+	−	++	+++	++	+	+
16	>10	+++	++	++	++	+	++	++	++	++	+	+
17	>10	+++	−	++	+	+	+	+++	++	++	+	−
1’	1	−	−	++	−	+	−	++	++	++	−	−
2’	2	−	−	++	−	+	−	++	++	++	−	−
3’	3	−	−	++	−	+	−	++	++	++	−	−

Note: Samples numbers 1–17: *C. reticulata* ‘Chachi’; 1′–3′: *C. reticulata* ‘Dahongpao’; +: the MS signal intensity of samples was 10^3^–10^4^; ++: the MS signal intensity of samples was 10^5^–10^6^; +++: the MS signal intensity of samples was 10^7^–10^8^ and −: undetected.

## Data Availability

The data supporting the results of this study are included in the present article and Supplementary Materials.

## Supplementary Materials

The following can be downloaded at https://www.mdpi.com/article/10.3390/foods13010008/s1, Table S1: Detailed samples information of *C. reticulata* ‘Chachi’ and *C. reticulata* ‘Dahongpao’. Table S2: Comparison of EIC retention time and fragmentation ions between compounds **1**–**12** and *C. reticulata* ‘Chachi’ under ESI-CID-MS. Figure S1: MS scan spectra of compounds **7**, **9**, **11** and **12** in LC-HR-MS. Figure S2: MS/MS spectra of MMA at *m*/*z* 166. Figure S3: EIC for protonated molecular weights of MMA at *m*/*z* 166, 3-hydroxyindole and *C. reticulata* ‘Chachi’ at *m*/*z* 134.06. Figure S4: Comparison of MRM chromatogram between compounds and *C. reticulata* ‘Dahongpao’. (A) isatin; (B) indirubin; (C) MDA; (D) *N*-methyl-3-hydroxyindole; (E) *N*-methylisatin.
