# Supplementary material for: Mass Spectrometry Rearrangement Ions and Metabolic Pathway-Based Discovery of Indole Derivatives during the Aging Process in Citrus reticulata ‘Chachi’
Source: Foods. 2023 Dec 19;13(1):8. doi: 10.3390/foods13010008 (PMC10778486; doi:10.3390/foods13010008)
Supplement: Supplementary file 1 [file foods-13-00008-s001.zip › foods-2721109-supplementary.pdf]

Mass spectrometry rearrangement ions and metabolic pathways-based discovery of indole derivatives during the aging process in *Citrus reticulata* 'Chachi'

Tian Li[1, 2], Ke Chen[2], Xiaoming Wang[2], Ying Wang[3, *], Yue Su[1, *], Yinlong Guo[2]

[1] *Institute of Interdisciplinary Integrative Medicine Research, Shanghai University of Traditional Chinese Medicine, 1200 Cailun Road, Shanghai 201203, China*

[2] *State Key Laboratory of Organometallic Chemistry and National Center for Organic Mass Spectrometry in Shanghai, Shanghai Institute of Organic Chemistry, Chinese Academy of Sciences, Shanghai 200032, China*

[3] *Institute for Control of Chinese Traditional Medicine and Ethnic Medicine, National Institutes for Food and Drug Control, No. 31 Huatuo Road, Daxing District, Beijing, 102629, China*

**Correspondence and requests for materials should be addressed to Y. S. (email: suyue@shutcm.edu.cn.)*

Supporting Material

Experimental Procedure **1**

Synthesis of compounds 7, 9, 11 and 12 1

Characterization of compounds 7, 11 and 12 .... **2**

Supplemental Tables and Figures **3**

Table S1 Detailed samples information of *C. reticulata* 'Chachi' and *C. reticulata* 'Dahongpao'... 3

Table S2. Comparison of EIC retention time and fragmentation ions between compounds 1-12 and *C. reticulata* 'Chachi' under ESI-CID-MS 5

Figure S1. MS scan spectra of compounds 7, 9, 11 and 12 in LC-HR-MS. 6

Figure S2. MS/MS spectra of MMA at *m/z* 166. 6

Figure S3. EIC for protonated molecular weights of MMA at *m/z* 166 ,3-hydroxyindole and *C. reticulata* 'Chachi' at *m/z*134.06 7

Figure S4. Comparison of MRM chromatogram between compounds and *C. reticulata* ' Dahongpao '. (A) isatin; (B) indirubin; (C) MDA; (D) *N*-methyl-3-hydroxyindole; (E) *N*-methylisatin 8

Experimental Procedure

Synthesis of compounds 7, 9, 11 and 12

Compound 7: To a well-stirred solution of 3-hydroxyindole (0.0248 g, 0.186 mmol) in THF (3 mL) at 0 °C was added sodium hydride (60% in mineral oil, 0.35 mmol). After 30 min stir in ice bath, the reaction flask was cooled again to 0 °C and CH_3I (0.0524 g, 0.369 mmol) was added dropwise. The reaction mixture was warmed to room temperature (rt) and allowed to stir until the reaction completed (monitored by TLC) and quenched with saturated aqueous NH_4Cl at 0 ºC (the amount of ice water was equivalent to the amount of reaction solution). The product was extracted with ethyl acetate (EtOAc, 3 × 20 mL) and washed once with saturated ammonium chloride and saturated salt water. Then it was dried over anhydrous Na_2SO_4. The organic phase was concentrated in vacuum to obtain the crude mixture which was further dissolved with dichloromethane (DCM), climbed the plate in 10% EtOAc/hexane and fractionated (5% methanol/DCM) to obtain the product.

Compound 9：A 10 mL Schlenk tube was charged with Pd $(OAc)_2$ (0.075 mmol), 1-methylindole (5 mmol), (diacetoxyiodo)benzene (10 mmol), and KOH (5 mmol). The reaction tube was purged with nitrogen. MeCN (20 mL) was then added to the reaction tube with a syringe. The Schlenk tube was placed in an oil bath and heated to 70 °C for 12 h and then cooled to rt. The mixture was then quenched by a sodium bisulfite solution (10 mL) and extracted by ethyl acetate (2 × 20 mL). The combined organic phases were dried over anhydrous Na_2SO_4, and concentrated under vacuum. After column chromatography (PE/ EtOAc = 99/1), a light green solid was obtained, which was hydrolyzed under alkaline conditions to obtain compound 9. Due to its instability and susceptibility to oxidation, nuclear magnetic resonance (NMR) data have not been obtained.

compounds 11 and 12: A 30 mL scintillation vial was charged with indigo or indirubin (1.0 equiv), Cs_2CO_3 (2.1 equiv), DMF (0.60 mL/mmol indigo) and CH_3I (4.0 equiv) under air. The vial was sealed, and the mixture was stirred at rt overnight. The resulting mixture was diluted with EtOAc, washed with brine (1x), H_2O (2x) and then brine (1x). The organic layer was dried over Na_2SO_4, and the filtrate was concentrated.

The crude product was purified with column chromatography (EtOAc/cyclohexane).

Characterization of compounds 7, 11 and 12

Compounds 7:

^{1}H NMR (400 MHz, $CDCl_3$) δ 7.74 (d, J = 8.0 Hz, 2H), 7.37 (d, J = 8.4 Hz, 2H), 7.30 (t, J = 7.6 Hz, 2H), 7.15 (t, J = 7.6 Hz, 2H), 3.80 (s, 6H), 3.58 (s, 6H).

Compounds 11:

^{1}H NMR (400 MHz, $CDCl_3$) δ 7.68 (d, J = 7.6 Hz, 2H), 7.51 (t, J = 7.6 Hz, 2H), 7.05 (d, J = 8.4 Hz, 2H), 7.01 (t, J = 7.6 Hz, 2H), 3.56 (s, 6H).

^{13}C NMR (100 MHz, $CDCl_3$) δ 185.2, 153.0, 135.0, 126.2, 123.8, 121.5, 121.1, 110.4, 36.5.

Compounds 12:

^{1}H NMR (400 MHz, $CDCl_3$) δ 8.59 (d, J = 8.0 Hz, 1H), 7.69 (d, J = 7.6 Hz, 1H), 7.55 (t, J = 8.0 Hz, 1H), 7.28 (d, J = 8.0 Hz, 1H), 7.08-7.01 (m, 3H), 6.82 (d, J = 7.6 Hz, 1H), 3.58 (s, 3H), 3.29 (s, 3H).

Supplemental Tables and Figures

Table S1 Detailed samples information of *C. reticulata* 'Chachi' and *C. reticulata* 'Dahongpao'.

Sample No.	Species	Origins
C. reticulata 'Chachi'-1	*Citrus reticulata* 'Chachi'	Chinese material processing plant, Guangdong, China
C. reticulata 'Chachi'-2	*Citrus reticulata* 'Chachi'	Xinhui, Jiangmen, China
C. reticulata 'Chachi'-3	*Citrus reticulata* 'Chachi'	Xinhui, Jiangmen, China
C. reticulata 'Chachi'-4	*Citrus reticulata* 'Chachi'	Xinhui, Jiangmen, China
C. reticulata 'Chachi'-5	*Citrus reticulata* 'Chachi'	Xinhui, Jiangmen, China
C. reticulata 'Chachi'-6	*Citrus reticulata* 'Chachi'	Xinbaotang Chenpi Co., Ltd
C. reticulata 'Chachi'-7	*Citrus reticulata* 'Chachi'	Xinbaotang Chenpi Co., Ltd
C. reticulata 'Chachi'-8	*Citrus reticulata* 'Chachi'	Chinese material processing plant, Guangdong, China
C. reticulata 'Chachi'-9	*Citrus reticulata* 'Chachi'	Xinhui, Jiangmen, China
C. reticulata 'Chachi'-10	*Citrus reticulata* 'Chachi'	Xinbaotang Chenpi Co., Ltd
C. reticulata 'Chachi'-11	*Citrus reticulata* 'Chachi'	'Chen Shengji 'Xinhui Chenpi
C. reticulata 'Chachi'-12	*Citrus reticulata* 'Chachi'	Xinhui, Jiangmen, China
C. reticulata 'Chachi'-13	*Citrus reticulata* 'Chachi'	Traditional Chinese Medicine Market, Zhangshu , China
C. reticulata 'Chachi'-14	*Citrus reticulata* 'Chachi'	Traditional Chinese Medicine Market, Zhangshu City, Jiangxi Province
C. reticulata 'Chachi'-15	*Citrus reticulata* 'Chachi'	Xinhui, Jiangmen, China

C. reticulata 'Chachi'-16	*Citrus reticulata* 'Chachi'	Xinhui, Jiangmen, China
C. reticulata 'Chachi'-17	*Citrus reticulata* 'Chachi'	'Chen Shengji 'Xinhui Chenpi
C. reticulata 'Dahongpao'-1	*Citrus reticulata* 'Dahongpao'	Zizhong, Neijiang, China
C. reticulata 'Dahongpao'-2	*Citrus reticulata* 'Dahongpao'	Zizhong, Neijiang, China
C. reticulata 'Dahongpao'-3	*Citrus reticulata* 'Dahongpao'	Enyang, Bazhong, China

Table S2. Comparison of EIC retention time and fragmentation ions between compounds 1-12 and *C. reticulata* 'Chachi' under ESI-CID-MS.

Compounds	retention time of EIC/min		fragment ions [M+H] $^+$	
	Reference substance	*C. reticulata* 'Chachi'	Reference substance	*C. reticulata* 'Chachi'
MMA	6.20	6.20	134.0, 116.0, 106.1, 91.0, 79.0, 77.0	134.0, 91.0, 116.0, 77.0, 106.1, 79.0, 89.0, 105.0
3-hydroxyindole	3.42	3.42	116.0, 106.1, 105.0, 91.0, 79.0, 77.0	77.0, 106.1, 105.0, 79.0, 91.0, 116.0, 52.0, 89.0
Isatin	3.39	3.39	92.0, 130.0, 102.0, 120.0, 65.0, 77.0	92.1, 120.0, 91.0, 65.0, 133, 102, 77, 130
Indigo	5.70	5.70	235.0, 219.0, 132.0, 217.0, 206.0, 245.0, 190.0	235.1, 206.0, 190.1, 132.0, 217.0, 245.0, 219.1
Indirubin	6.08	6.08	219.0, 235.0, 217.0, 245.0, 190.0, 132.0	235.1, 217.0, 165.1, 245, 219.0, 206.0
N, N', O, O' -Tetramethyl-leuko-indigo	8.11	8.11	306.1, 291.1, 275.1, 247.1, 175.1, 160.1	306.1, 291.1, 275.0, 247.0, 175.1, 160.0
MDA	2.77	2.77	120.0, 148.0, 91.0, 118.0, 77.0, 105.1, 130.0, 133.0	133.0, 134.0, 91.0, 162.1, 106.0, 79.0, 120.0, 77.0, 105.0
N-methyl-3-hydroxyindole	3.99	3.99	133.0, 79.0, 105.0, 120.0, 77.0, 130.0	92.0, 91.0, 120.0, 133.0, 77.0, 105.1, 104.1, 106.1
N-methylisatin	3.83	3.83	116.0, 106.0, 79.0, 65.0, 77.0, 134.0, 144.0	116.0, 65.0, 134.0, 106.0, 77.1, 120.0, 144.0
N, N'- demethylindigo	6.15	6.15	132.0, 276.0, 146.1, 247.1, 120.1, 172.1, 263.1	247.1, 132.0, 146.0, 276.0, 209.0, 79.0
N, N' - demethylindirubin	6.53	6.53	247.1, 276.1, 146.1, 132.0, 263.1, 158.1	247.1, 276.1, 146.1, 132.0, 263.1, 158.1

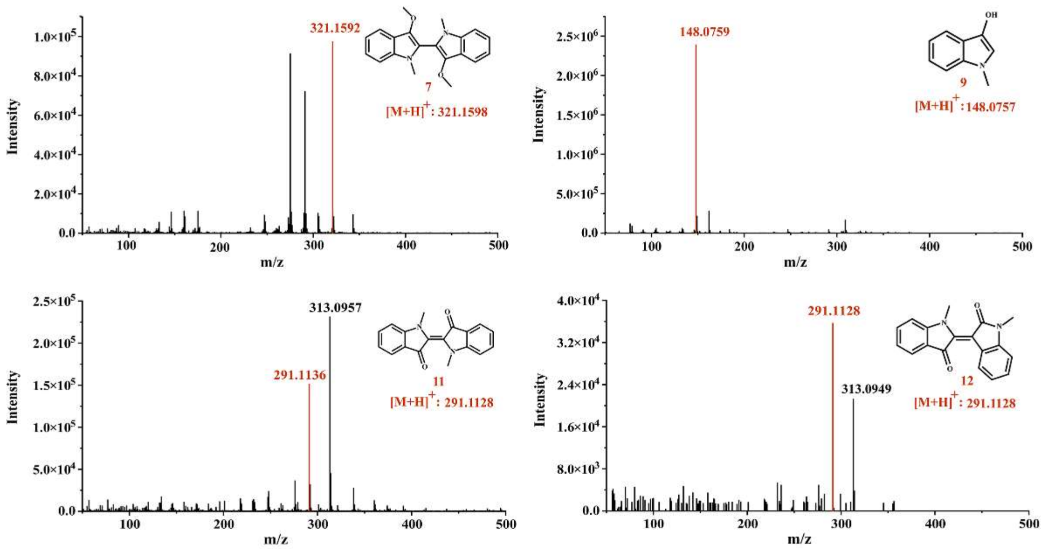

Figure S1. MS scan spectra of compounds 7, 9, 11 and 12 in LC-HR-MS.

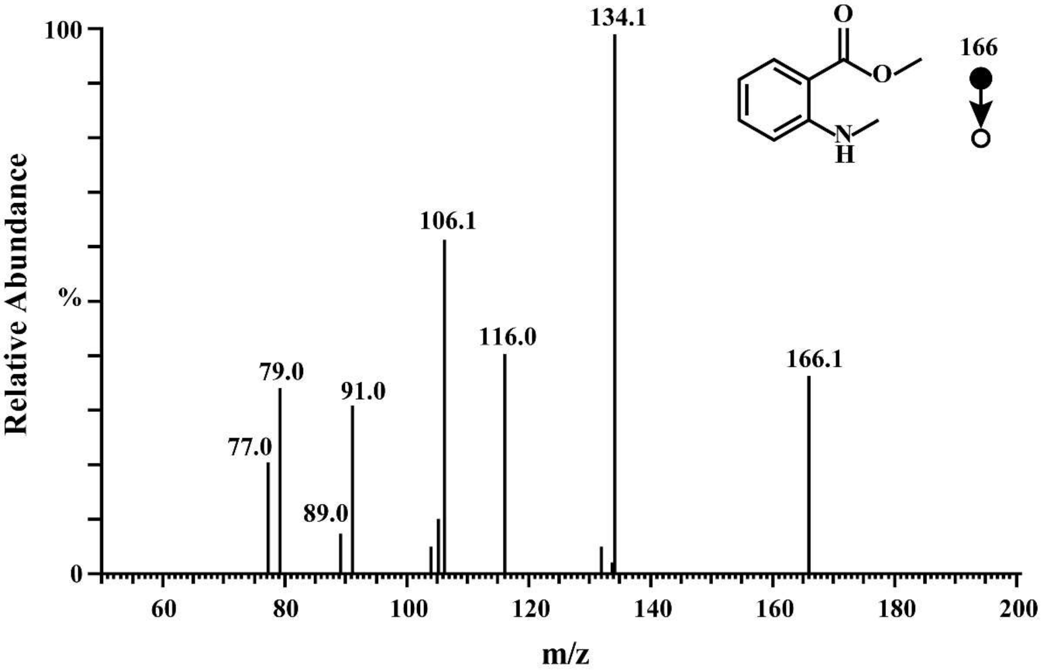

Figure S2. MS/MS spectra of MMA at *m/z* 166.

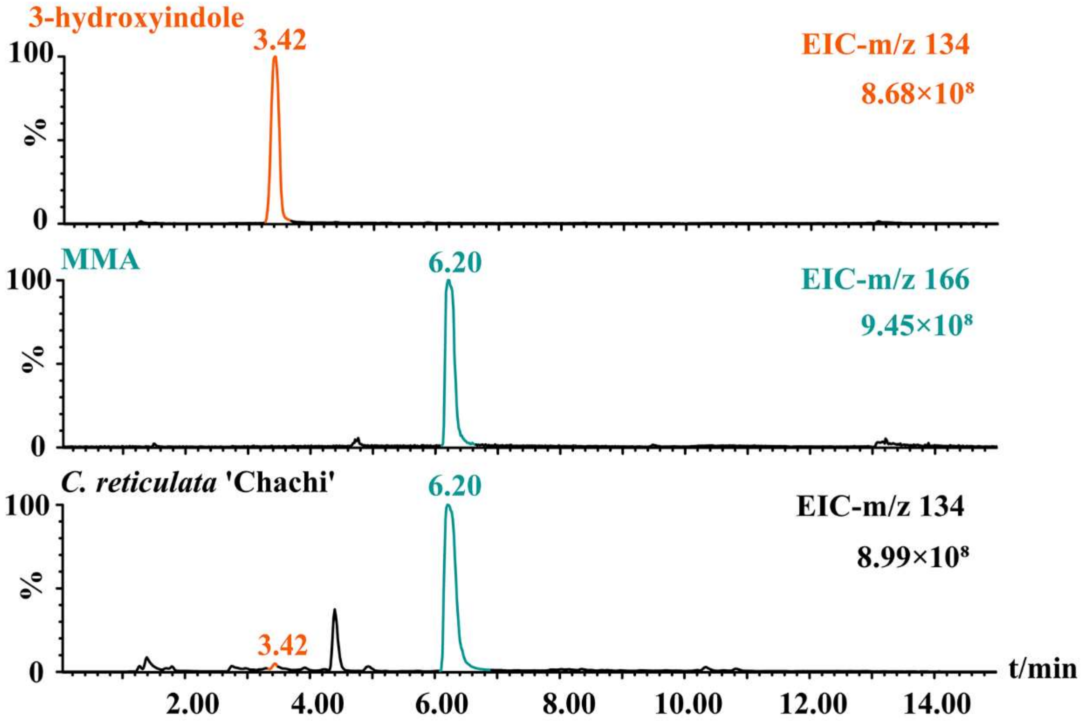

Figure S3. EIC for protonated molecular weights of MMA at *m/z* 166 ,3-hydroxyindole and *C. reticulata* 'Chachi' at *m/z*134.06

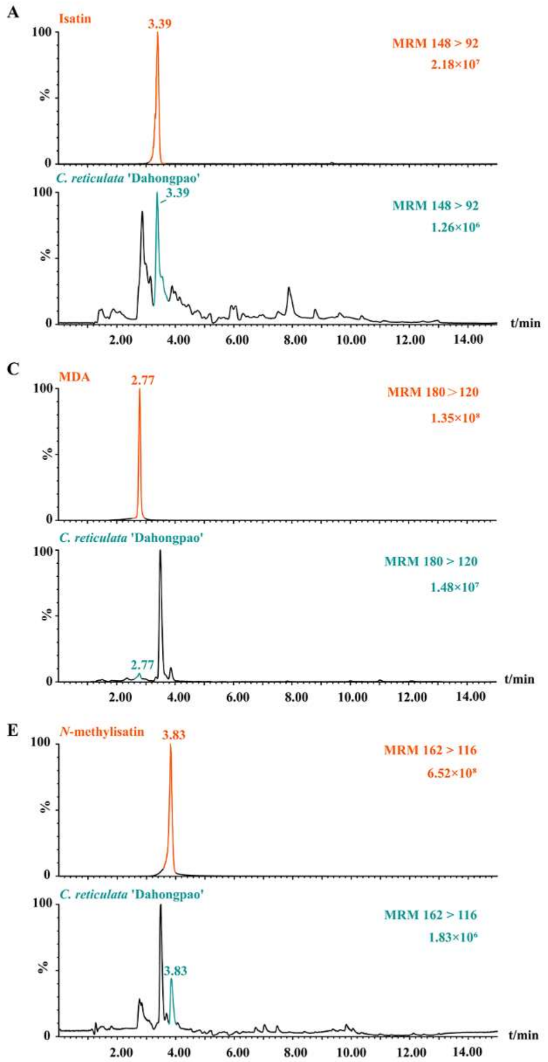

Figure S4. Comparison of MRM chromatogram between compounds and *C. reticulata* ' Dahongpao '. (A) isatin; (B) indirubin; (C) MDA; (D) *N*-methyl-3-hydroxyindole; (E) *N*-methylisatin.
